# A Possible Case of Varicella Zoster Virus (VZV) Meningoencephalitis in an Immunocompetent Host

**DOI:** 10.7759/cureus.26539

**Published:** 2022-07-03

**Authors:** Sanya Goswami, Rohan Goyal, John DeLury

**Affiliations:** 1 Internal Medicine, State University of New York Downstate Medical Center, Brooklyn, USA; 2 Infectious Diseases, State University of New York Downstate Medical Center, Brooklyn, USA

**Keywords:** varicella zoster virus encephalitis, varicella zoster virus, immunocompetent, meningoencephalitis, biofire film array

## Abstract

The herpes zoster infection occurs in the United States, particularly targeting those who are immunocompromised, and can present with many manifestations including encephalitis. Instances of varicella zoster virus (VZV) encephalitis in immunocompetent patients have been rarely reported, but such diagnoses are becoming more frequent as detection of VZV has improved with the adoption of molecular diagnostic panels such as the BioFire Film Array meningitis panel (Salt Lake City, USA). Here, we present an interesting case of acute meningoencephalitis in an immunocompetent adult female without dermatomal neuralgia or cutaneous lesions attributable to VZV. Given many inconsistencies between the patient’s presentation and the positive polymerase chain reaction (PCR) result for VZV, we suspected our patient was infected with an undetected organism while possibly simultaneously shedding previously acquired VZV. As molecular diagnostic panels are increasingly used and have greatly improved detection of rarer etiologies of disease, we encourage clinicians to interpret results with caution.

## Introduction

Varicella zoster virus (VZV) is a member of the alphaherpesviridae family. Primary infection most often occurs during childhood but has become rare in developed countries since the introduction of the VZV vaccine. Herpes zoster, also known as shingles, results from reactivation of latent VZV in the dorsal root ganglia or trigeminal ganglia, and affects 1.2 million individuals annually in the United States. Viral reactivation is often precipitated by an immunocompromised state caused typically by aging or impaired T-cell mediated immunity [1]. The most common findings include rash in the dermatomal distribution of the sensory ganglion within three to four days and acute neuritis, which presents as a painful, often deep “throbbing” sensation that can precede the rash by a couple of days. About 1% to 15% of patients with herpes zoster develop post herpetic neuralgia, with a higher incidence in the immunocompromised population. In some cases, dermatomal neuralgia can develop without any identifiable skin manifestations throughout the clinical course, termed zoster sine herpete [1,2]. Other less common clinical presentations include herpes zoster ophthalmicus, acute retinal necrosis, Ramsay-Hunt syndrome, and myelitis [3-5].

Herpes-zoster-associated encephalitis, or VZV encephalitis, typically presents with altered mental status and delirium within days following the vesicular rash eruption but can also occur prior to the onset of the rash. The involved nerve cells exhibit inflammation and hemorrhagic necrosis, and this damage is also reflected by the typical neuropathic pain associated with herpes zoster [6]. This pathophysiology is very similar to the encephalitis caused by the closely related herpes simplex virus (HSV). Although CSF findings may be relatively similar between the two encephalitides, with viral pattern and elevated red blood cell count due to hemorrhagic necrosis of affected neurons, VZV encephalitis does not classically display a cerebral lobar preference, whereas HSV encephalitis typically involves the frontal and temporal lobes [7]. Lobar involvement can be observed as enhancement on MRI brain or as EEG changes including focal slowing or periodic discharges [8].

VZV encephalitis in immunocompetent hosts has been reported in the presence of both the typical vesicular rash of VZV as well as zoster sine herpete [9,10]. However, to the knowledge of this report’s authors, VZV encephalitis in the absence of any dermatomal signs or symptoms has not. Although this is a rare diagnosis, there still have been increasing numbers of cases involving immunocompetent patients secondary to physicians increasingly utilizing high-sensitivity molecular panels to confirm diagnosis [11]. Here, we present a rare case of acute meningoencephalitis in an immunocompetent adult female without dermatomal neuralgia or cutaneous lesions attributable to VZV.

## Case presentation

A 61-year-old female with no known medical conditions, on no home medications, presented to the emergency department (ED) for a one-week history of persistent worsening right-sided headache associated with subjective fevers, myalgias, decreased appetite, occasional non-bloody non-bilious emesis, cough, and flu-like symptoms. Per the patient’s husband, the patient displayed unusual behaviors over the course of the week, including asking her husband basic questions about his job and daily schedule. The patient had previously sought care previously at another ED twice for similar symptoms where she was given intravenous fluids (IVF) and pain medication; she was discharged with antitussive medication. At home, the patient experienced short episodes of disorientation after which she returned to baseline. At presentation, the patient reported feeling restless with a mild headache after dinner; she took a nap and then woke up feeling “delusional,” agitated, and with a severe headache intractable to ibuprofen. She was subsequently brought to our ED. In the ED, the patient was able to identify those around her and provide a limited history and the remainder was obtained by the family at bedside. The patient was up to date on all vaccines apart from SARS-CoV-2. The patient’s history was otherwise notable for ownership of a daycare center and chicken pox during childhood; there was no history of recent travel or toxic habits. At this time, review of symptoms was negative for chest pain, shortness of breath, abdominal pain, fatigue, and joint pain. 

On presentation, the patient’s vital signs were temperature of 97.6ºF, heart rate of 83/min, blood pressure of 152/83 mmHg, and respiratory rate of 20/min. Physical exam was remarkable for diaphoresis, confusion, and agitation. Pupils were round and equally reactive to light, gag reflex was intact, and Kernig and Brudzinski signs were negative. The remainder of the neurologic exam was limited due to the patient’s inability to follow commands, and the remaining cardiac, pulmonary, abdominal, and musculoskeletal exam were within normal limits. Laboratory studies were notable for serum potassium of 5.6 and white blood cell (WBC) count of 3.19 K/μL (neutrophil count of 1.68 K/μL, no bands).

Several hours later, while waiting in the ED, the patient was observed to have a generalized tonic-clonic seizure and was subsequently found to be agitated, confused, thrashing, and screaming. Her speech was noted to be unintelligible, and she was unable to follow commands. Pupils were equally round and reactive, but only opened to pain. The remainder of the physical exam was unchanged. The patient was sedated with midazolam. Laboratory studies were notable for WBC count of 7.53 K/μL, bicarbonate 16 mmol/L, anion gap 24 mEq/L, venous pH 7.19, and lactate 8.7 mmol/L. Erythrocyte sedimentation rate (ESR) and C-reactive protein (CRP) were not obtained. Point-of-care influenza A and B RNA were negative. CT head was negative for acute intracranial pathology but showed calvarial thinning and small extra-axial fluid collections along the right parietal lobe and bilateral parafalcine frontal lobes. Chest x-ray was unremarkable except for cardiomegaly. The patient was empirically treated with intravenous 10 mg/kg acyclovir every eight hours, 1.25 g vancomycin every 12 hours, 2 g ampicillin every four hours, and 2g ceftriaxone every 12 hours. She was also started on 750 mg levetiracetam every 12 hours for seizure prophylaxis. Lumbar puncture was then performed, with the fourth tube showing glucose of 71, WBC count of 102, lymphocyte count of 89, polymorphic mononuclear cell count of 2, and protein of 92, consistent with a viral/aseptic pattern. CSF Gram stain showed no organisms. CSF studies of BioFire FilmArray meningitis/encephalitis panel (Salt Lake City, USA), encephalopathy, autoimmune/paraneoplastic evaluation (ENC2) panel, HSV 1/2 PCR, cytomegalovirus (CMV) PCR, human polyomavirus 2 (JC virus), Lyme antibodies, cryptococcal antigen, venereal disease research laboratory (VDRL), rapid plasma reagin (RPR), angiotensin-converting enzymes (ACE) level, and tuberculosis, as well as serum West Nile virus immunoglobulin G (IgG) and immunoglobulin M (IgM) all returned as negative except for positive VZV polymerase chain reaction (PCR) found via the CSF meningitis/encephalitis panel.

**Figure 1:**
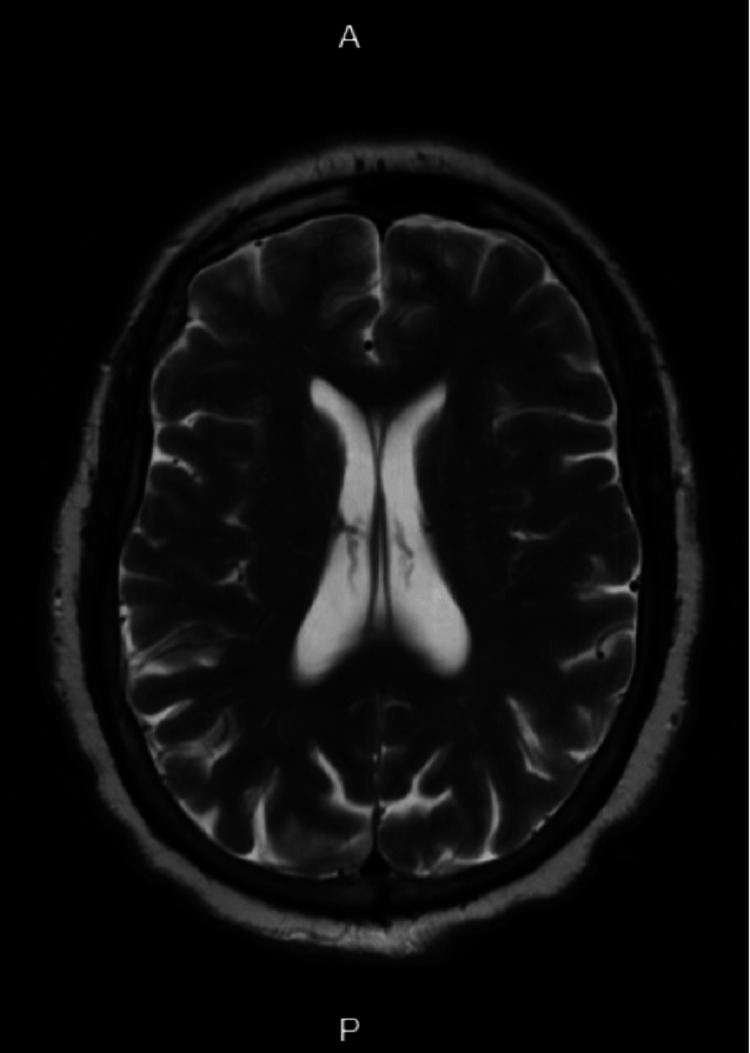
MRI brain A: Anterior; P: Posterior

The patient was then admitted to the medicine service and was closely followed by the neurology and infectious diseases teams. The patient’s mental status dramatically improved overnight. The following morning, the patient had returned to her baseline, was hemodynamically stable, and reported no new symptoms. On exam, she was alert and oriented to person, place, and time, appropriately answered questions, followed three-step commands, performed basic calculations, and had intact three-word recall after five minutes. MRI brain was performed, only showing cerebral atrophy and minimal chronic microvascular ischemic changes; there was no parenchymal edema, discrete fluid collection, or abnormal enhancement noted (see Figure 1). EEG showed diffuse delta-theta slowing with superimposed focal slowing over the bitemporal regions. Diffuse slowing was noted to be consistent with moderate to severe diffuse cerebral dysfunction which may be seen in meningoencephalitis; focal superimposed slowing in the bitemporal lobes was noted to suggest focal dysfunction.

Based on diagnostic findings and resolution after empiric acyclovir, antibiotics, and levetiracetam, the provisional diagnosis of VZV meningoencephalitis was made with a high degree of suspicion for other viral etiology, due to the absence of skin manifestations or known reason for immunocompromised status and rapid recovery inconsistent with a typical VZV encephalitis course. Throughout the remainder of the admission, the patient’s mental status remained at baseline with no further seizure episodes. Labs remained unremarkable, and antibiotic therapy was discontinued.

The patient was discharged with valacyclovir 1 g thrice daily for a 14-day course, levetiracetam 750 mg twice daily, and nifedipine extended-release 30 mg once daily. In addition, the patient was discharged with close outpatient neurology and infectious disease follow-up appointments. At the patient’s outpatient infectious diseases appointment after completing the course of medications, the patient reported complete resolution of headaches and no seizure-like activity. Neurologic and dermatologic exams were unremarkable.

## Discussion

This is an interesting case of acute meningoencephalitis in an immunocompetent adult female that rapidly resolved after presentation. Although only VZV PCR returned positive, the history and clinical course were felt to be inconsistent with classic VZV encephalitis as reports of VZV encephalitis without dermatomal involvement remain extremely rare. The association with a flu-like illness is much more typical of a new acute infection, which is also consistent with the patient’s exposure to a daycare facility. The rapid resolution of symptoms overnight after initiation of acyclovir therapy and absence of enhancement on brain MRI are also inconsistent with classical VZV encephalitis. As the diagnostic detection of VZV in CSF has improved with broader use of molecular diagnostic panels that rapidly test for many infectious agents, it has become increasingly necessary for physicians to determine how best to interpret positive findings when inconsistent with typical presentations of disease. 

In the case of central nervous system infections, the BioFire FilmArray meningitis/encephalitis panel has become commonly used to test cerebrospinal fluid for bacterial, viral, and fungal organisms. Specifically, this panel tests for *Escherichia coli* K1, *Haemophilus influenzae*, *Listeria monocytogenes*, *Neisseria meningitidis*, *Streptococcus agalactiae*, *S. pneumoniae*, CMV, enterovirus (EV), HSV-1, HSV-2, human herpesvirus-6 (HHV-6), human parechovirus (hPeV), VZV, *Cryptococcus neoformans*, and *Cryptococcus gatti*. This panel offers the advantage of testing for many pathogens within hours and allows rapid treatment initiation for often fatal diagnoses [12]. Per a 2020 meta-analysis, the panel was estimated to have a sensitivity of approximately 90% and a specificity of 97% [13]. After the adoption of the meningitis/encephalitis panel, increased diagnostic detection of VZV has been reported in multiple hospitals [11].

Given the high specificity of the meningitis/encephalitis panel, it is possible but unlikely that the positive VZV result was a false positive. Nevertheless, the patient’s clinical course and presentation remain highly inconsistent with VZV encephalitis. One possible alternative cause could be asymptomatic shedding of VZV while experiencing encephalitis due to another etiology. 0.39% of immunocompetent individuals with prior VZV infection have been reported to asymptomatically shed the virus at a given time; this is potentially a mechanism by which the virus is able to remain present within the host organism for years after the primary infection [14]. Therefore, regarding this patient’s case, we suspect either a highly atypical presentation of VZV encephalitis in an immunocompetent patient or infection with an agent that escaped diagnostic detection in our workup, where the positive VZV PCR result can be explained by simultaneous asymptomatic shedding of the virus originally acquired during childhood.

Difficulties correlating other positive results on the meningitis/encephalitis panel with clinical presentations have been previously documented. For example, the HHV-6 virus is often noted to return positive without clinical significance. In our clinical experience, positive HHV-6 results in immunocompetent patients with altered mental status have also contributed to diagnostic confusion. In a study in 2018, 25% of positive meningitis/encephalitis panel results were positive for HHV-6, but the majority of these were not associated with a detectable plasma viral load; these results have been replicated [15,16]. Although HHV-6, like VZV, demonstrates subclinical latent reactivation that can manifest as asymptomatic viral shedding, some of these results can also be explained by direct amplification of chromosomally integrated viral DNA [17]. These clinically insignificant, but technically accurate, positive results should be differentiated from false positive results, which have mainly been noted with bacterial agents including *S. pneumoniae* and *S. agalactiae* [13].

## Conclusions

In addition to documenting a unique case of a positive VZV result on the meningitis/encephalitis panel in an immunocompetent host with rapid resolution of encephalitis symptoms, we hope to clarify the role of molecular diagnostic panels in identifying less obvious etiologies. We strongly recommend that clinicians consider a broad differential when clinical pictures do not correlate with lab results. Although molecular diagnostic panels have greatly improved the detection of rarer etiologies of disease, we encourage clinicians to interpret results with caution as molecular diagnostic panels continue to become more common.
